# Pattern of Blood Pressure Indices among the Residents of a Rural Community in South East Nigeria

**DOI:** 10.4061/2011/621074

**Published:** 2011-10-25

**Authors:** B. J. C. Onwubere, E. C. Ejim, C. I. Okafor, A. Emehel, A. U. Mbah, U. Onyia, S. Mendis

**Affiliations:** ^1^Department of Medicine, University of Nigeria Teaching Hospital, PMB 01129, Enugu, Nigeria; ^2^Department of Community Medicine, University of Nigeria Teaching Hospital, PMB 01129, Enugu, Nigeria; ^3^Cardiovascular Disease, World Health Organization, Geneva, Switzerland

## Abstract

Cardiovascular diseases (CVDs) are the main causes of death in industrialized countries, and are significant causes of morbidity and mortality in sub-Saharan Africa. Hypertension is the most common cardiovascular disease in Nigerians, and the risk of CVD associated with hypertension is independent of other risk factors. Despite the high level of awareness of its presence in the developed countries, the level of control is still poor. CVDs tend to be commoner in urban settlements, and it has been hypothesized that rural sub-Saharan Africa is at an early stage of epidemiological transition from communicable to non-communicable diseases (NCD) because of the gradual adoption of unhealthy lifestyles. This study aimed at describing the pattern of blood pressure indices among the hypertensive residents of a rural community in South East Nigeria. A total of 858 individuals comprising 247 males and 611 females took part in the study. 46.4% of the subjects had hypertension. Hypertension was commoner in the males (50.2% vs. 44.8%) (*χ* ^2^(1) = 1.484; *P* = 0.223). The males were significantly older and heavier than the females while the females had higher mean values of BMI and WC. The prevalence of hypertension is becoming alarmingly high in the rural communities of sub-Saharan Africa.

## 1. Introduction

 Cardiovascular disease (CVD) is the main cause of death in industrialized countries and is increasingly recognized as a significant cause of morbidity and mortality in sub-Saharan Africa including Nigeria [1, 2]. Globally, hypertension is the most common cardiovascular disease even among Nigerians [3]. The risk of CVD associated with hypertension is independent of other risk factors [4]. Part of the worrisome issues surrounding these CVDs is that of poor control in over two-thirds of the individuals affected by these conditions despite the high level of awareness of their presence in the developed countries [5]. This high burden of poor control definitely contributes to the mortality pattern seen globally. These diseases tend to be more common in urban settlements, and it has been hypothesized that rural sub-Saharan Africa is at an early stage of epidemiological transition from communicable to noncommunicable diseases (NCDs) because of the gradual adoption of unhealthy lifestyles [6, 7]. These lifestyles characterized by increasing intake of high calorie-dense foods and physical inactivity result in obesity [8–10].

Though the current emphasis now is on both systolic blood pressure (SBP) and diastolic blood pressure (DBP) based on current weight of evidence, it does appear that the risk associated with them is still more with SBP. Before now, DBP had been the attention of the medical community until facts on the risk associated with SBP began to emerge, one of which was that from Framingham Heart Study in 1971 [11–13].

Several studies have compared rural and urban populations or attempted to describe cardiovascular risk factors in rural communities [14–16]. For instance, in Ghana, earlier studies revealed a hypertension prevalence of 4.5% among rural dwellers while, in Nigeria, the prevalence of hypertension was found to be 10% in rural areas [17, 18].

## 2. Subjects and Methods

This study was carried out in a rural Igbo community in Ezeagu local government area of Enugu state. The community—Imezi Owa—is located about 20 km from Enugu, the capital city of Enugu State in south east Nigeria. Its inhabitants are mainly elderly men and women, their children, and some young and middle-aged women. The population is estimated to be about 28,808 people based on 2005 population census figures. The community is predominantly dominated by children and their young/middle-aged mothers as well as the elderly men and women, most of who have retired from active work. The young and middle-aged men live in the cities where they are more gainfully employed and visit their families at the rural areas periodically. The few young and middle-aged men who reside in these rural areas are mostly subsistent farmers and work in the farms from the early hours of the morning till late in the evening, and they are usually not available for these community-based studies.

All the adults aged 40–70 years from randomly selected households totaling eight hundred were invited for the study. Out of about 1,219 subjects invited, 858 people reported at the health centres for data collection giving a response rate of 70.4%. Informed consent was duly obtained from all the participants using the information sheet and consent forms. The research protocol was approved by the Ethics Committee of the University of Nigeria Teaching Hospital Ituku Ozalla, Enugu.

## 3. Data Collection

All measurements were conducted by two trained physicians and one nurse between 8 AM and 10 AM at designated health centres. Questionnaires were administered by the physicians and data obtained included age, gender, and occupation.

Systemic blood pressure was measured using a standard mercury sphygmomanometer on the left arm after 5-minute rest using the appropriate size with the subject in the sitting position. The first and fifth phases of Korotkoff sounds were used for systolic (SBP) and diastolic blood pressures (DBP) respectively. Two independent measurements were obtained with a minimum interval of one minute [19]. The mean of the two blood pressure readings was used for each subject in this study. All the blood pressure measurements for all the subjects were done by the same doctor with the same mercury sphygmomanometer, while the second doctor did all the biochemical examinations with the Accutrend equipment on all the subjects.

 Anthropometric measurements including height, weight, waist, and hip circumferences were done by the nurse. Height was measured without shoes to the nearest centimeter using a ruler attached to the wall, while weight was measured to the nearest 0.1 kg on an electronic scale with the subject wearing light outdoor clothing and no shoes. Waist circumference was measured at the highest point of the iliac crest with the subject in light clothing.

## 4. Blood Tests

Fasting blood samples for dry chemistry were collected under aseptic procedures from a finger puncture. The participants had been earlier instructed during the selection of households to observe a minimum of eight-hour overnight fast to be eligible to participate in the study. Compliance with this instruction was ascertained from each participant before samples were collected. Dry chemistry tests were used to measure total cholesterol (TC) and fasting blood glucose (FBG) level using the Accutrend GC System (Accutrend GC, Roche Diagnostics, Germany). Measuring ranges of the device were 1.1–33.3 mmol/L and 3.88–7.76 mmol/L for glucose and total cholesterol, respectively. The Accutrend GC System does not measure LDL cholesterol, HDL cholesterol or VLDL cholesterol; hence these lipid parameters were not assessed. Precision for glucose was <3% and for cholesterol <5%. Accuracy of the Accutrend GC system for glucose test was ±5% compared to a hexokinase protein-free precipitate method and for cholesterol was ±5% compared with cholesterol oxidase/P-aminophenazone (CHOP-PAP) method. Results for the glucose were obtained within 12 seconds and 180 seconds for cholesterol test. Measuring principle of the device was reflectance photometry.

Sample volumes needed for the device were one drop of blood for cholesterol and one drop of blood for glucose applied directly from the fingertip. The Accu-chek softclix prolancing device was used in the study for the finger prick.

## 5. Definition of Risk Factors

Hypertension was defined as systolic blood pressure ≥140 mmHg and/or diastolic blood pressure ≥90 mmHg [20] or being on pharmacological treatment for hypertension. The different grades of hypertension were defined as follows: mild: 140–159/90–99 mmHg, moderate: 160–179/100–109 mmHg, and severe hypertension: ≥180/110 mmHg. Subjects were classified as having hypertension using either the SBP only, DBP only or both. 

Participants were diagnosed to have diabetes mellitus (DM) if they had fasting blood glucose level (FBG) ≥7 mmol/L or reported a history of diabetes or use of glucose-lowering drugs [21]. Subjects with impaired fasting glycaemia (IFG) were defined by FBG ranging from 6.1 to 6.99 mmol/L. Subjects found to have DM or IFG were regarded to have abnormal glucose tolerance (dysglycemia). Overweight and generalized obesity were defined as body mass index (BMI) ≥25 and 30 kg/m^2^, respectively. Abdominal obesity was defined as waist circumference of ≥102 cm in men and ≥88 cm in women [22]. High cholesterol was defined as greater than or equal to 6.2 mmol/L [23].

Statistical analysis was done using the Personal Computer (PC) analytical software Statistical Package for Social Sciences (SPSS Inc, Chicago Ill) version 17. Results were expressed as either mean values (standard deviation) or proportions, and comparison for statistical significance was by *t* test for continuous variables or chi-square analysis for categorical variables. Pearson's correlation was done to determine the degree of relationship between some quantitative variables while multiple stepwise linear regression analysis was used to determine the significant predictors of SBP and DBP. The level of significance level was set at *P* ≤ 0.05.

## 6. Results

A total of 858 individuals made up of 247 (28.8%) males and 611 (71.2%) females took part in the study with 398 (46.4%) subjects defined as having hypertension. The characteristics of all the subjects are shown in Table 1. Mean indices of obesity (BMI, waist circumference, and waist-to-hip ratio) were higher among the hypertensive subjects. 

As shown in Table 2, the subjects with hypertension consisted of 124 (31.2%) males and 274 (68.8%) females. No significant differences were observed in the gender distribution of hypertension, mean blood pressure, and biochemical parameters. The male hypertensive subjects were significantly older than the females while the female subjects had higher mean values of BMI and WC.

**Figure 1:**
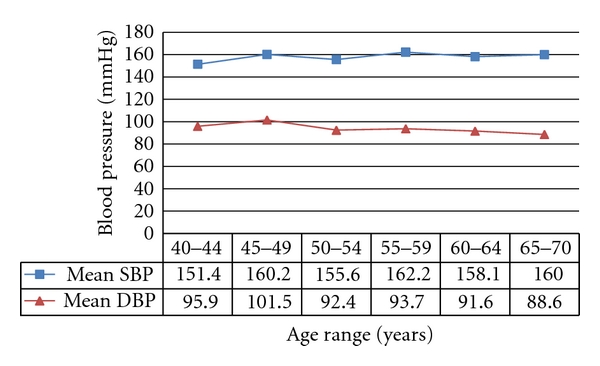
Trend of mean SBP and mean DBP with age.

Among these hypertensive subjects, hypercholesterolaemia was seen in 13 (3.3%) subjects while elevated fasting blood glucose profile was observed in 19 (4.8%) subjects made up of 11 subjects with impaired fasting glycaemia and eight subjects with diabetes mellitus. Central obesity was present in 141 (35.4%) subjects. Mean SBP and DBP varied between the age groups but these did not maintain a linear increase or decrease across the age groups, respectively (Figure 1). Findings from regression analysis with fitting of regression lines (diagram not shown), however, revealed that SBP and DBP tended to increase and decrease with age, respectively.

**Figure 2:**
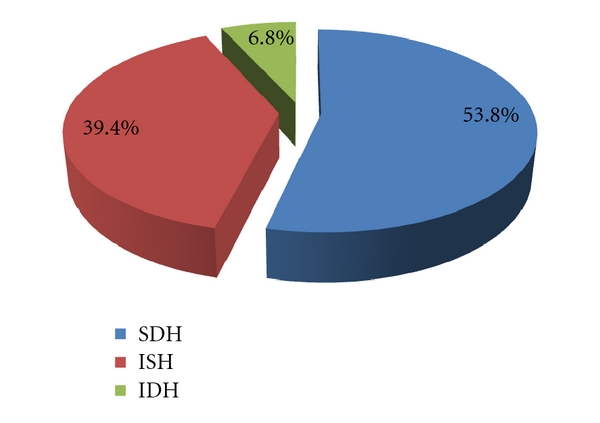
Definition of hypertension among the subjects using SBP or DBP or both.

 Over half of the 398 subjects were defined as having hypertension using systolic-diastolic hypertension (SDH) while the rest were defined using either isolated systolic (ISH) or diastolic (IDH) hypertension (Figure 2). 

**Figure 3:**
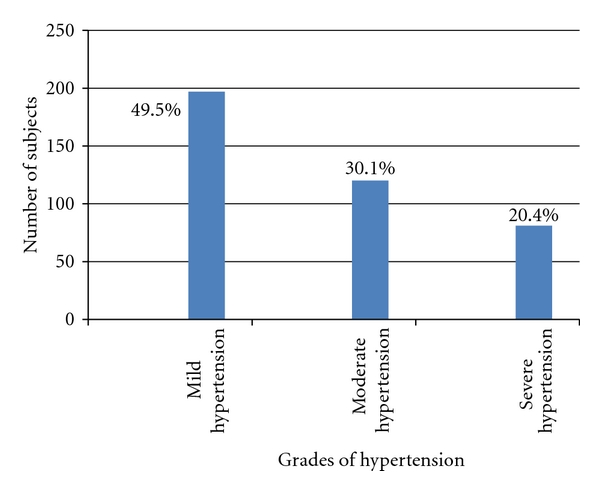
Grades of hypertension among the hypertensive subjects (% representation was inserted).

Mild hypertension was the most common grade of hypertension among the hypertensive patients (Figure 3). Mild, moderate and severe hypertension were observed in 197, 120, and 81 subjects, respectively. Optimal blood pressure was seen in 203 (44.1%) subjects among the 460 nonhypertensive subjects while the remaining 257 subjects had borderline blood pressure.

Evaluation of the most common pattern of blood pressure elevation among the different grades of hypertension showed that elevation of SBP was the most common pattern in those with mild hypertension (112 (56.9%) whereas elevation of both SBP and DBP was the most common pattern in those with moderate (80; 66.7%) and severe (70; 86.4%) hypertension (*χ* ^2^(4) = 79.0; *P* < 0.0001). 

Correlation analysis of SBP and DBP with some clinical characteristics showed some degree of correlation between the variables but the degree was very weak (Table 3). While most of the variables correlated significantly with DBP, only DBP correlated significantly with SBP.

A stepwise linear regression analysis to determine the predictors of systolic and diastolic blood pressures revealed diastolic blood pressure and age as the only significant predictors of SBP while age, SBP and waist circumference significantly predicted DBP (Table 4). 

## 7. Discussion

The majority of the subjects who responded to the invitation for participation in this study were females. This is likely due to the characteristics of the traditional African society where males are the major bread winners for their families and live in the cities while their wives and children live in the villages with the grandparents. These and other characteristics are highlighted in Section 2. 

The prevalence of hypertension as found in this study was high (about 46%). This is higher than that found in Gambia (6.8%) [6] but close to that documented among the general population in Latvia (46.1%) and among a market population in Enugu (about 40%) by Dzerve et al. [24] and Ulasi et al. [25]. The low prevalence found in the Gambian population may be due to the blood pressure definition of ≥160/95 mmHg used in the study [6]. The high prevalence found in this study is quite close to that found in Enugu [25] but the populations are not quite comparable as the market population could be described as a mixed population. This is so because the market is attended on daily basis not only by those residents in Enugu city but also by people who reside in the surrounding suburbs. Irrespective of these differences, the findings from Gambia [6] still established the pattern of higher prevalence of cardiovascular diseases in urban dwellers. The burden of other cardiovascular risk factors was quite low among the hypertensive patients in this study. The prevalence of IFG was low compared to 14% found among hypertensive subjects attending the Diabetes Clinic of UNTH, Enugu [26]. These diabetic subjects also demonstrated higher mean indices of obesity as was the pattern observed in this study thus establishing the possible association of obesity with CVDs even among rural dwellers. Among all the other CVD risk factors, central obesity was the most common in our study. Hypercholesterolaemia occurred in a very small proportion of the hypertensive subjects but no significant difference between hypertensive and nonhypertensive, male and female subjects was noted. Notwithstanding these, serum cholesterol has been reported to affect blood pressure regulation [27]. 

Similar to the study by Banegas et al. [28], SBP constituted a greater community burden than DBP in this study. SBP was also more common in those >50 years of age when compared to DBP and greater proportion of subjects were upstaged in this study by SBP than DBP. The burden of SBP and DBP in those <50 years was 5.1% and 27.5%, respectively. Over the years, emphasis has been laid on either SBP or DBP in terms of associated risk but the JNC-VI/WHO-ISH guideline/classification tends to have assigned relatively similar roles to both SBP and DBP but the morbidity and mortality risk associated with both indices still tends to tilt more to SBP [11–13]. As documented in this study, over 50% of hypertensive subjects were staged using both indices. 

Few variables were found to significantly predict hypertension among our subjects. Each of the indices, however, was also found to be a significant predictor of the other. Ageing was common as a predictor of both SBP and DBP in this study. Age is known to significantly influence the pattern of blood pressure; hence SBP tends to increase with advancing age as a result of loss of arterial compliance while DBP tends to flatten out or decrease after 50 years of age as was observed in this study [29]. The decrease in compliance results in higher systolic pressures. An increase in peripheral resistance is also known to result in elevations of diastolic blood pressures whereas the loss of elasticity of large vessels causes a reduction. With increasing age therefore, the net effect of these counteracting forces may result in a normal or near-normal diastolic pressure [30, 31]. The trend of DBP hence may depend on the predominant force. 

Central obesity represented by waist circumference was the other predictor of DBP. Obesity is a common CVD risk factor in Nigerian hypertensive patients [32]. These predictors were similar to those also found to predict hypertension in both rural and urban Gambian communities [6].

## 8. Conclusion

The prevalence of hypertension is becoming alarmingly high in the rural communities contrary to the existing trend of rarity noted many years ago despite the traditional way of life of these inhabitants. It was also observed that systolic-diastolic as well as isolated systolic hypertension is dominant in these subjects and they are therefore likely to be exposed to high risks of hypertension-related morbidity and mortality. Ageing and obesity are also significant predictors of hypertension among rural dwellers.

##  Recommendation

We recommend that in view of the increasing trend in prevalence, there is the need to institute educational programmes to guard against possible epidemiologic transition among these rural dwellers. It is to be noted that of all the predictors of hypertension observed in this study, while obesity is modifiable, age is not.

##  Limitations

Though this is a population-based study, the cross-sectional design of this study requires that the findings be interpreted cautiously. The use of two blood pressure readings taken at one sitting may also have influenced the blood pressure readings obtained and used to classify the subjects.

## Figures and Tables

**Table 1:** Clinical Characteristics of all the subjects by their hypertensive status.

Variable	Presence of hypertension		*P*-value*
	Yes (*n* = 398)	No (*n* = 460)	
Age (yrs)	61.2 (9.3)	58.6 (10.2)	<0.0001
Height (m)	1.56 (0.08)	1.56 (0.07)	0.779
Weight (kg)	57.6 (13.5)	55.9 (12.9)	0.06
Body mass index (Kg/m^2^)	23.6 (5.3)	22.8 (4.6)	0.019
Waist circumference (cm)	85.9 (12.6)	83.5 (12.7)	0.006
Waist to Hip ratio	0.88 (0.07)	0.87 (0.06)	0.011
Systolic blood pressure (mmHg)	158.4 (19.7)	118.6 (12.8)	<0.0001
Diastolic blood pressure (mmHg)	91.4 (14.1)	70.9 (9.3)	<0.0001
Fasting blood glucose (mmol/L)	4.6 (1.5)	4.6 (1.8)	0.989
Total cholesterol (mmol/L)^†^	4.6 (0.8)	4.6 (0.9)	0.972

^†^The sample sizes of the overall, men, and women groups for total cholesterol estimation were 292 and 301 subjects, respectively. The rest of the subjects had low values (i.e., <3.88 mmol/L) based on the measuring range of the machine. *P value between hypertensive and non hypertensive subjects.

**Table 2:** Clinical Characteristics of the hypertensive subjects by gender.

Variable	Men (*n* = 124)	Women (*n* = 274)	*P* value
Proportion with hypertension (%)	50.2	44.8	0.223
Age (yrs)	62.6 (9.4)	60.6 (9.3)	0.046
Height (m)	1.62 (0.07)	1.53 (0.06)	<0.0001
Weight (kg)	58.4 (11.1)	57.2 (14.5)	0.38
Body Mass Index (Kg/m^2^)	22.2 (3.9)	24.3 (5.7)	<0.0001
Waist circumference (cm)	83.9 (9.7)	86.8 (13.6)	0.015
Waist to Hip ratio	0.9 (0.05)	0.88 (0.09)	0.002
Systolic blood pressure (mmHg)	156 (18.2)	159.2 (20.3)	0.269
Diastolic blood pressure (mmHg)	91.3 (13.1)	91.4 (14.6)	0.942
Fasting blood glucose (mmol/L)	4.7 (1.9)	4.6 (1.3)	0.531
Total cholesterol (mmol/L)^†^	4.6 (1.0)	4.6 (0.7)	0.612

^†^The sample sizes of the overall, men, and women groups for total cholesterol estimation were 292, 72, and 220 subjects, respectively. The remaining 106 subjects had low values (i.e., <3.88 mmol/L) based on the measuring range of the machine.

**Table 3:** Pearson's correlation analysis of SBP and DBP with some clinical characteristics.

Variables	Systolic blood pressure		Diastolic blood pressure	
	*R*	*P* value*	*r*	*P* value*
Age	0.05	0.321	−0.235	<0.0001
Weight	0.061	0.226	0.203	<0.0001
BMI	0.088	0.077	0.196	<0.0001
WC	0.061	0.221	0.152	0.002
WHR	−0.08	0.110	−0.024	0.634
SBP	1	—	0.436	<0.0001
FBG	−0.047	0.348	0.039	0.434
TC	0.087	0.135	0.061	0.295

*Significance was 2-tailed.

**Table 4:** Regression analysis of systolic and diastolic blood pressure with variables.

		Unstandardized coefficients			
	Predictors	*B*	Standard error	*T*	*P* value
SBP	Constant	76.835	9.268	8.290	<0.0001
	DBP (mmHg)	0.664	0.064	10.364	<0.0001
	Age (yrs)	0.339	0.096	3.527	<0.0001

DBP	Constant	48.019	9.169	5.237	<0.0001
	SBP (mmHg)	0.325	0.037	8.855	<0.0001
	Age (yrs)	−0.294	0.077	−3.792	<0.0001
	WC (cm)	0.122	0.056	2.188	0.029
